# Cardiac Time Intervals by Tissue Doppler Imaging M-Mode: Normal Values and Association with Established Echocardiographic and Invasive Measures of Systolic and Diastolic Function

**DOI:** 10.1371/journal.pone.0153636

**Published:** 2016-04-19

**Authors:** Tor Biering-Sørensen, Rasmus Mogelvang, Martina Chantal de Knegt, Flemming Javier Olsen, Søren Galatius, Jan Skov Jensen

**Affiliations:** 1 Department of Cardiology, Herlev and Gentofte Hospital, University of Copenhagen, Copenhagen, Denmark; 2 The Copenhagen City Heart Study, Frederiksberg Hospital, University of Copenhagen, Copenhagen, Denmark; 3 Institute of Clinical Medicine, Faculty of Health Sciences, University of Copenhagen, Copenhagen, Denmark; Sapienza University of Rome, ITALY

## Abstract

**Purpose:**

To define normal values of the cardiac time intervals obtained by tissue Doppler imaging (TDI) M-mode through the mitral valve (MV). Furthermore, to evaluate the association of the myocardial performance index (MPI) obtained by TDI M-mode (MPI_TDI_) and the conventional method of obtaining MPI (MPI_Conv_), with established echocardiographic and invasive measures of systolic and diastolic function.

**Methods:**

In a large community based population study (n = 974), where all are free of any cardiovascular disease and cardiovascular risk factors, cardiac time intervals, including isovolumic relaxation time (IVRT), isovolumic contraction time (IVCT), and ejection time (ET) were obtained by TDI M-mode through the MV. IVCT/ET, IVRT/ET and the MPI ((IVRT+IVCT)/ET) were calculated. We also included a validation population (n = 44) of patients who underwent left heart catheterization and had the MPI_TDI_ and MPI_Conv_ measured.

**Results:**

IVRT, IVRT/ET and MPI all increased significantly with increasing age in both genders (p<0.001 for all). IVCT, ET, IVRT/ET, and MPI differed significantly between males and females, displaying that women, in general exhibit better cardiac function. MPI_TDI_ was significantly associated with invasive (dP/dt max) and echocardiographic measures of systolic (LVEF, global longitudinal strain and global strainrate s) and diastolic function (e’, global strainrate e)(p<0.05 for all), whereas MPI_Conv_ was significantly associated with LVEF, e’ and global strainrate e (p<0.05 for all).

**Conclusion:**

Normal values of cardiac time intervals differed between genders and deteriorated with increasing age. The MPI_TDI_ (but not MPI_Conv_) is associated with most invasive and established echocardiographic measures of systolic and diastolic function.

## Introduction

Cardiac time intervals are sensitive markers of cardiac dysfunction, even when it goes unrecognized by conventional echocardiography[1–4]. These subtle impairments in cardiac function, which are revealed by disturbed cardiac time intervals, have previously been demonstrated to contain prognostic information incremental to the conventional echocardiographic parameters[4–8]. The great potential of the cardiac time intervals to capture signs of cardiac dysfunction was already demonstrated half a century ago[9]. Since then, cardiac ultrasound machines have evolved and new methods of obtaining cardiac time intervals have been suggested. The most common method of obtaining cardiac time intervals by echocardiography is from velocity curves obtained from pulsed-Doppler echocardiography of the left ventricular (LV) outflow tract and mitral valve (MV) inflow[10,11]. It is hereby possible to calculate the index of combined systolic and diastolic performance and is known as the myocardial performance index (MPI_Conv_)[10,11]. Additionally, using the newer echocardiographic modality, tissue Doppler imaging (TDI) which provides regional myocardial velocity curves and, thereby, regional cardiac time intervals[12] and MPI (MPI_Regional_), has in recent years been an attractive method in the obtainment of cardiac time intervals[13–15]. The two aforementioned methods of obtaining cardiac time intervals have, however, several limitations which have been a limiting factor in the clinical applicability of these methods until now. A novel method has recently evolved where global cardiac time intervals are obtained through evaluation of MV movement through the cardiac cycle by using a simple color TDI M-mode analysis[4,7,8,16–19]. Using color TDI M-mode through the MV to estimate cardiac time intervals is an improved method, with better precision and reproducibility[17,18], reflecting global cardiac time intervals and eliminating beat-to-beat variation and regional differences.

Nevertheless, clear definitions of normal and pathological cardiac time intervals, including their normal values, have to be established before we can use these in a clinical setting in order to be able to identify miniscule levels of impaired cardiac function. In addition, the association between this novel method of obtaining MPI (MPI_TDI_) and established echocardiographic and invasive measures of systolic and diastolic function has to be evaluated. The aim of this study was, therefore, to define normal values of cardiac time intervals obtained by TDI M-mode through the MV in a large sample obtained from the general population without known cardiovascular disease and cardiovascular risk factors. A further aim of this study was to evaluate the association of MPI_TDI_ (the novel method of obtaining MPI) and MPI_conv_ (the conventional method of obtaining MPI) as described by Tei and colleagues[10,11] with established echocardiographic and invasive measures of systolic and diastolic function.

## Method

### Study population

Our study population consists of individuals without hypertension, diabetes, ischemic heart disease (IHD), heart failure (CHF) or atrial fibrillation who are included in the echocardiographic sub study of the fourth Copenhagen City Heart Study (Study group A)[4,8,18,20], and an invasive validation group consisting of patients suspected of ischemic heart disease who underwent coronary angiography, left heart catheterization and echocardiography (Study group B).

#### Population contributing to the normal values (group A)

This population includes all participants from the fourth Copenhagen City Heart Study examination 2001–2003, who had an echocardiographic examination, including TDI, performed and who were free from any cardiovascular disease and cardiovascular risk factor (n = 974)(Tables 1 and 2).

**Table 1:** Population characteristics in participants without cardiovascular disease or cardiovascular risk factors obtained from the derivation cohort (group A, n = 974).

	Women (n = 553)	Men (n = 421)	*p value*
Age (years)	50±15	49±14	*0*.*69*
Systolic Blood Pressure (mmHg)	118±12	123±10	*<0*.*001*
Diastolic Blood Pressure (mmHg)	72±9	75±8	*<0*.*001*
Heart Rate (beats per minute)	65±10	64±11	*0*.*038*
Cholesterol (mmol/l)	5.3±1.1	5.3±1.1	*0*.*93*
BMI (kg/m^2^)	23.7±3.2	25.1±3.0	*<0*.*001*
LVEF < 50%	0.2%	0.7%	*0*.*19*
Diastolic dysfunction	0.8%	0.3%	*0*.*30*
LVIDd (cm)	4.6±0.4	5.1±0.5	*<0*.*001*
LAD (cm)	3.2±0.3	3.5±0.4	*<0*.*001*
E/A ratio < 1	26%	25%	*0*.*74*
DT (ms)	158±32	169±40	*<0*.*001*
E/e’	8.9 (7.6–10.5)	8.5 (7.4–10.2)	*0*.*06*
LVMI (g/m^2^)	72.4 (64.4–81.7)	88.1 (77.0–99.9)	*<0*.*001*

BMI = Body Mass Index; LVEF = Left Ventricular Ejection Fraction; LVMI = Left Ventricular Mass Index; LVIDd = left ventricular dimension in end-diastole; LAD = Left atrium diameter in end-systole; E = peak transmitral early diastolic inflow velocity; A = peak transmitral late diastolic inflow velocity; DT = deceleration time of early diastolic inflow; e’ = average peak early diastolic longitudinal mitral annular velocity determined by color tissue Doppler imaging obtained from all six myocardial walls.

**Table 2:** Normal values of the cardiac time intervals by TDI M-mode in healthy participants stratified according to age category and gender obtained from the derivation cohort (group A, n = 974).

	Women (n = 553)					Men (n = 421)					
	Overall (n = 553)	Age category 20 to 39 (n = 150)	Age category 40 to 59 (n = 252)	Age category 60 or above (n = 151)	*p-value for age category difference*	Overall (n = 421)	Age category 20 to 39 (n = 101)	Age category 40 to 59 (n = 210)	Age category 60 or above (n = 110)	*p-value for age category difference*	*p-value for gender difference*
**IVRT (ms)**	92 (20)	78 (16)	93 (16)	106 (20)	*<0*.*001*	94 (20)	78 (15)	95 (16)	109 (18)	*<0*.*001*	*0*.*13*
**IVCT (ms)**	36 (13)	32 (12)	37 (12)	38 (14)	*<0*.*001*	34 (11)	35 (11)	33 (11)	36 (12)	*0*.*07*	*0*.012*
**ET (ms)**	293 (21)	291 (19)	296 (20)	289 (23)	*0*.*001*	281 (24)	284 (19)	280 (23)	279 (28)	*0*.*34*	*<0*.001*
**IVRT/ET**	0.32 (0.07)	0.27 (0.05)	0.31 (0.06)	0.37 (0.07)	*<0*.*001*	0.34 (0.08)	0.28 (0.06)	0.34 (0.06)	0.40 (0.08)	*<0*.*001*	*<0*.001*
**IVCT/ET**	0.12 (0.05)	0.11 (0.04)	0.13 (0.04)	0.13 (0.05)	*<0*.*001*	0.12 (0.04)	0.13 (0.04)	0.12 (0.04)	0.13 (0.05)	*0*.*10*	*0*.*61*
**MPI**	0.44 (0.10)	0.38 (0.08)	0.44 (0.08)	0.51 (0.10)	*<0*.*001*	0.46 (0.10)	0.40 (0.08)	0.46 (0.09)	0.53 (0.10)	*<0*.*001*	*0*.002*

Numbers in parenthesis are standard deviations. BMI = Body Mass Index; LVMI = Left Ventricular Mass Index; LVIDd = left ventricular dimension in end-diastole; LAD = Left atrium diameter in end-systole; DT = deceleration time of early diastolic inflow; IVCT = Isovolumic Contraction Time; IVRT = Isovolumic Relaxation Time; ET = Ejection Time; MPI = Myocardial Performance Index

* remained statistical significantly different between the genders after multivariable adjustment for age, BMI, heart rate, systolic and diastolic blood pressure, LVMI, LVIDd, LAD, and DT. Age category difference was determined by ANOVA.

#### Invasive validation population (group B)

The validation population includes patients suspected of ischemic heart disease who underwent coronary angiography and left heart catheterization (n = 44) at Department of Cardiology, Gentofte Hospital, University of Copenhagen between April 2009 and February 2011. JSJ and SG were the treating physicians performing coronary angiography in group B.

All participants gave written informed consent, and the study was performed in accordance with the second Helsinki Declaration and approved by the regional ethics committee for the region of Copenhagen and the Danish data protection agency.

### Invasive measurements

In group B, left heart catheterization was performed. Left ventricular (LV) pressures were measured, and an echocardiogram was performed according to a standardized protocol. Invasively measured pressure curves were obtained using 5 French pigtail catheters placed in the LV chamber and the pressure set and transducer were calibrated. Measurements were obtained over at least three cardiac cycles and saved as hard copies and digitalized in relation to this study. The LV pressure curves were digitalized using Dagra Version 2.0.12.35924. In all cardiac cycles the rate of LV pressure rise in early systole (dP/dt max) and the rate of LV pressure decline in early diastole (dP/dt min) were measured and averaged over all the cardiac cycles. In addition to dP/dt max and dP/dt min, the time constant of LV isovolumic pressure decline (tau) was measured.

### Echocardiography

All echocardiograms were obtained using Vivid ultrasound systems (GE Healthcare, Horten, Norway) with a 2.5-MHz transducer. All participants were examined with conventional two-dimensional echocardiography and color TDI. All echocardiograms were stored and analyzed offline with commercially available software (EchoPac, GE Medical, Horten, Norway).

#### Conventional echocardiography

In group A [8,18], regional function was evaluated by the standard 16-segment model, as suggested by the American Society of Echocardiography[21]. LV ejection fraction (LVEF) was evaluated by 1 observer on the basis of the wall motion index score. Additional in group A, diastolic dysfunction was determined by E/A-ratio and DT, and defined as DT < 140 ms and E/A_<50 years_ > 2.5, E/A_50–70 years_ > 2, or E/A_>70 years_ > 1.5, respectively[4,8]. In group B, LVEF was obtained using modified biplane Simpson’s method[21].

The myocardial thickness and the dimensions of the LV in end-diastole (LVIDd) and the left atrium in end-systole (LAD) were measured. LV mass index (LVMI) was calculated as the anatomic mass[21] divided by body surface area[22]. Pulsed wave Doppler at the apical view was utilized to record mitral inflow between the tips of the MVs. Peak velocity of early (E) and atrial (A) diastolic filling and deceleration time of the E-wave (DT) were measured and the E/A-ratio was calculated. In group B, pulsed-wave TDI tracings were obtained with the range gate placed at the septal and lateral mitral annular segments in the 4-chamber view. The peak longitudinal early diastolic (e’) velocity was measured and the average was calculated from the lateral and septal velocities and used to obtain the E/e’ ratio. Furthermore, left atrial volume was estimated by the biplane area-length method and divided with BSA creating the Left Atrial Volume Index (LAVI). Impaired systolic function was defined as an LVEF below 50% and impaired diastolic function as e’ below 9 cm/sec and/or an LAVI of 34 mL/m^2^ or above.

Additionally, in group B time intervals were measured using the method described by Tei and colleagues[10,11]. The interval from MV closing to opening was determined as the period from the end to onset of mitral inflow (A to E time) obtained from the pulsed-wave Doppler at the apical position. LV ejection time (ET) was determined as the period from onset to end of LV outflow. MPI_Conv_ was calculated as: (A to E time–ET)/ET[10,11].

#### Two-dimensional strain echocardiography

In group B, Two-dimensional strain echocardiography (2DSE) was performed from the apical 4-chamber, 2-chamber and apical long-axis view (mean 79 frames/s, standard deviation 18 frames/s). By speckle tracking, the endocardial border was traced in end systole. The integrity of speckle tracking was automatically detected and visually ascertained. In case of poor tracking, the region of interest tracing was readjusted. Global longitudinal strain (GLS) was measured in all three apical views and averaged to provide GLS. Furthermore, in each three apical views, global longitudinal systolic strain rate (GL SR s) and global longitudinal early diastolic strain rate (GL SR e) were measured, and averaged to provide global estimates.

#### Cardiac time intervals obtained by color TDI M-mode

In group A [8,18], color TDI tracings were obtained with the range gate placed at the lateral, septal, inferior, anterior, posterior and anterior septal mitral annular segments in the 4-, 2- and 3-chamber view. In group A e’ was measured by color TDI and the average was calculated from the lateral, septal, inferior, anterior, posterior and anterior septal velocities and used to obtain E/e’ ratio.

**Fig 1:**
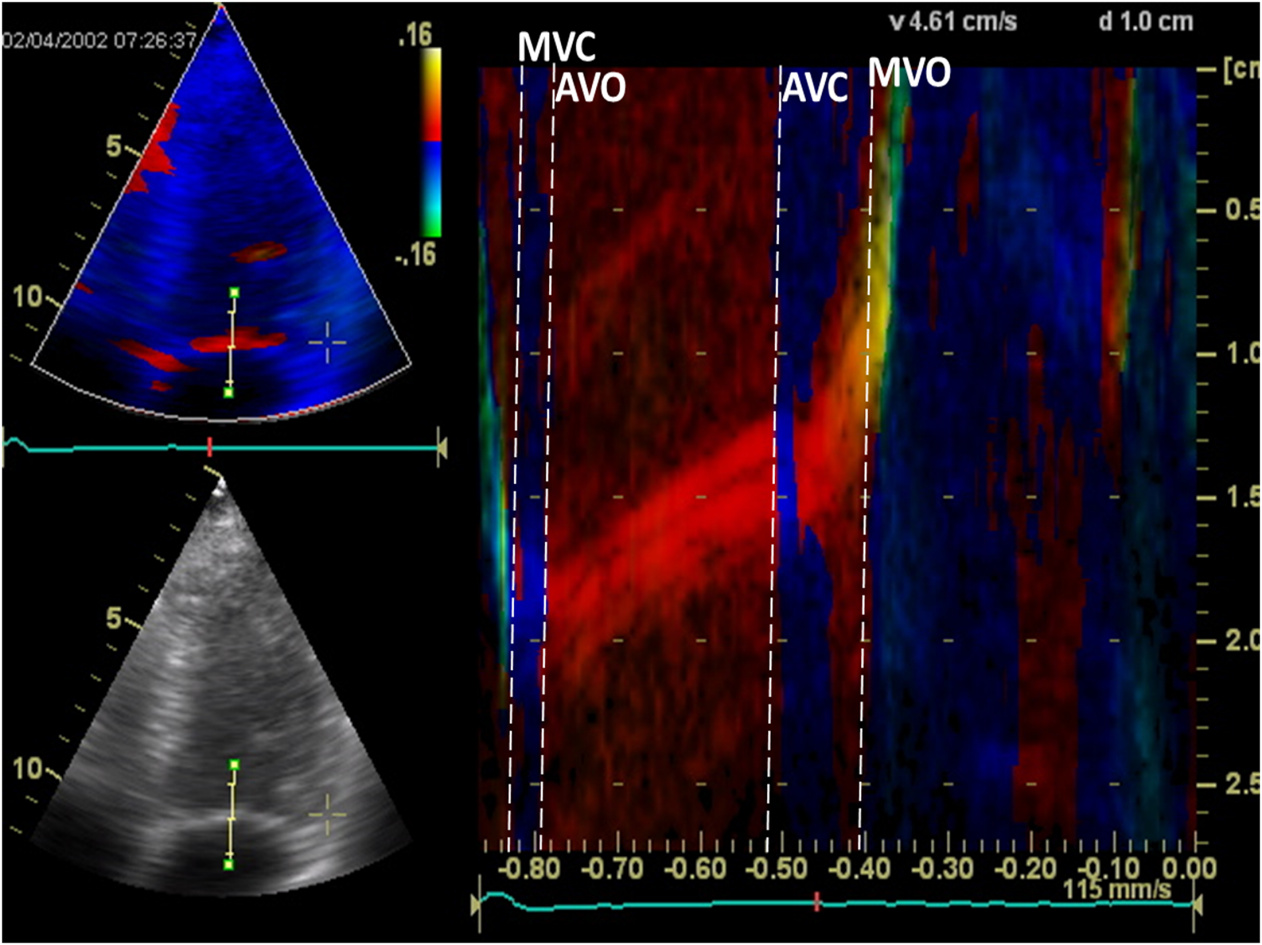
Cardiac time intervals assessed by a color tissue Doppler imaging (TDI) M-mode line through the mitral leaflet. Left: Four-chamber gray-scale (bottom) and color TDI (top) views in end-systole displaying the position of the M-mode line used for measuring the cardiac time intervals. Right: Color diagram of the TDI M-mode line through the mitral leaflet. MV = Mitral Valve; MVC = MV Closing; AVO = Aortic Valve Opening; AVC = Aortic Valve Closure; MVO = MV Opening.

In both group A [8,18] and group B, cardiac time intervals were obtained by placing a 2–4 cm straight M-mode line through the septal half of the MV in the color TDI 4-chamber view and cardiac time intervals were measured directly from the color diagram[7,8,18] (Fig 1). The IVCT was defined as the time interval from MV closure (MVC), determined by the color shift from blue/turquoise to red at end-diastole, to aortic valve opening (AVO) determined by the color shift from blue to red (Fig 1). ET was defined as the time interval from AVO to aortic valve closing (AVC), determined by the color shift from red to blue at end systole (Fig 1). IVRT was defined as the time interval from AVC to MV opening (MVO), determined by the color shift from red-orange to yellow (Fig 1). The method has previously been validated [7,17,18], and has been demonstrated to be superior to the conventional method of obtaining cardiac time intervals[17,18]. Furthermore, we have previously demonstrated high reproducibility of the method in this present study population[18]. Both isovolumic time intervals were divided with ET creating IVRT/ET and IVCT/ET, respectively, and MPI was calculated as the sum of the two ((IVRT+IVCT)/ET).

### Statistics

Proportions were compared using χ^2^-test, continuous Gaussian distributed variables with Student’s t-test or Mann-Whitney test if non-Gaussian distributed. The association between cardiac time intervals, age and gender was tested by univariable and multivariable regression analyses including significant confounders detected from the baseline differences between genders (Table 1). Linearity, variance homogeneity, and the assumption of normality were tested with plots of residuals. Trends were analyzed by linear regression analyses and departure from linearity was assessed by simultaneous assessment of linear and quadratic effects. Non-Gaussian distributed continues variables (LVMI and E/e’) were categorised into quartiles when included in the models. In Table 2 the age category difference was determined by ANOVA. A p-value ≤ 0.05 in 2-sided test was considered statistically significant. All analyses were performed with STATA Statistics/Data analysis, SE 12.0 (StataCorp, Texas,USA).

## Results

### Normal values of cardiac time intervals in healthy participants stratified by age and gender

**Fig 2:**
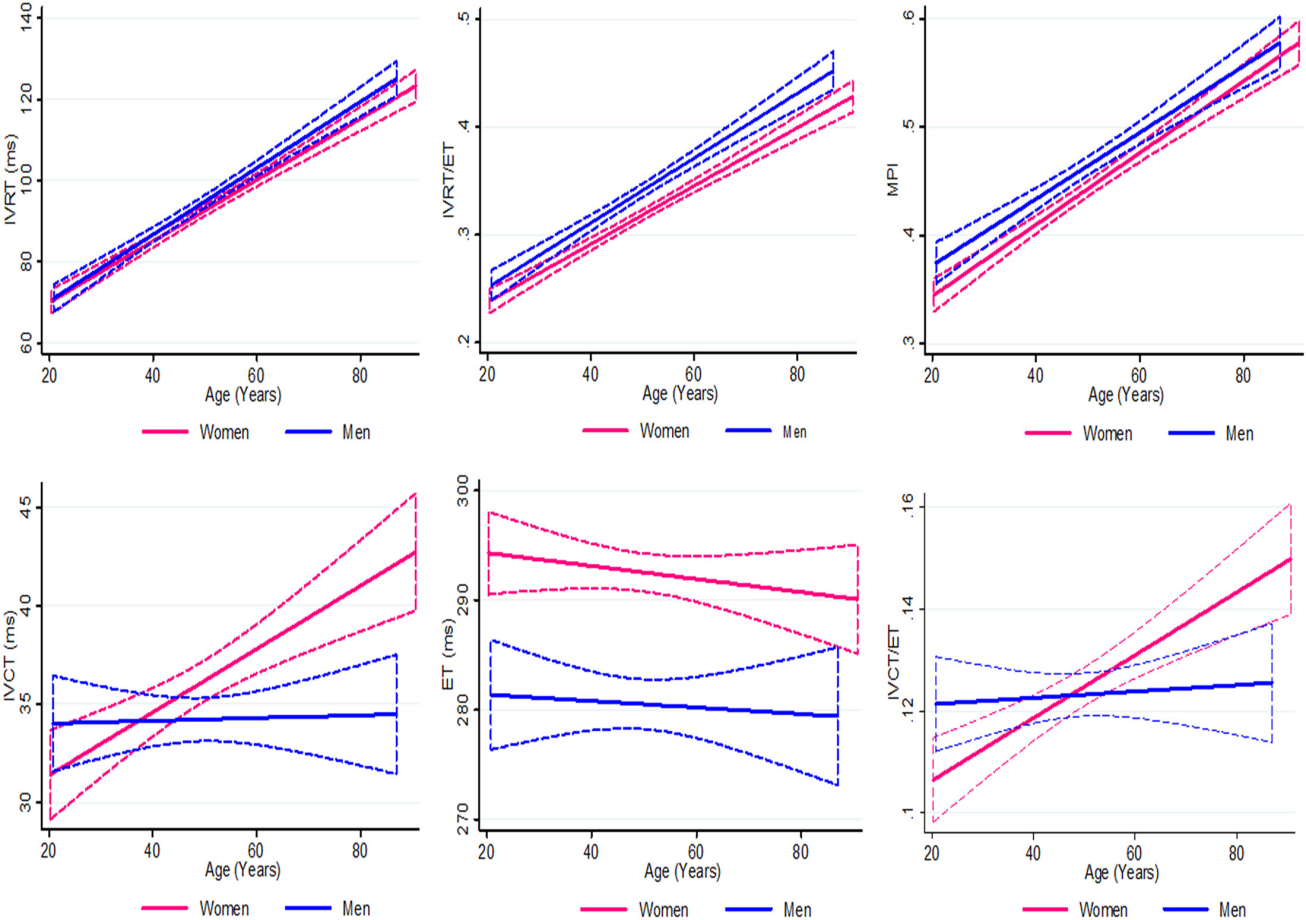
The expected cardiac time intervals according to age and gender in participants without cardiovascular disease or cardiovascular risk factors. Depicting the expected values of the cardiac time intervals with increasing age for participants stratified according to gender. Dotted curves indicate 95% confidence intervals. IVCT = Isovolumic Contraction Time; IVRT = Isovolumic Relaxation Time; ET = Ejection Time; MPI = Myocardial Performance Index.

In participants without hypertension, diabetes, IHD, CHF or atrial fibrillation, normal values of cardiac time intervals were assessed. The population that contributed to the normal values consisted of 974 participants, 553 women and 421 men, (Table 1). The study participants were stratified according to age (20 to 39, 40 to 59, and ≥ 60 years) and gender (Table 2 and Fig 2).

Gender significantly modified the relationship between age and two of the cardiac time intervals (IVCT and IVCT/ET) which is visualized in Fig 2 (p for interaction = 0.004 for IVCT; p for interaction = 0.005 for IVCT/ET). IVRT, IVRT/ET and MPI all increased significantly with increasing age in both genders (p<0.001 for all)(Table 2 and Fig 2). IVCT and IVCT/ET increased statistically significantly with increasing age in females but not in males (Table 2 and Fig 2). ET decreased with increasing age in both genders; however, this decline was not statistically significant (Table 2 and Fig 2). IVCT, ET, IVRT/ET, and MPI differed significantly between male and female gender. These gender differences remained statistically significant after multivariable adjustment for all other baseline differences between genders reported in Table 1.

### The association between MPI_TDI_ and MPI_Conv_, and invasive measures of systolic and diastolic function

**Fig 3:**
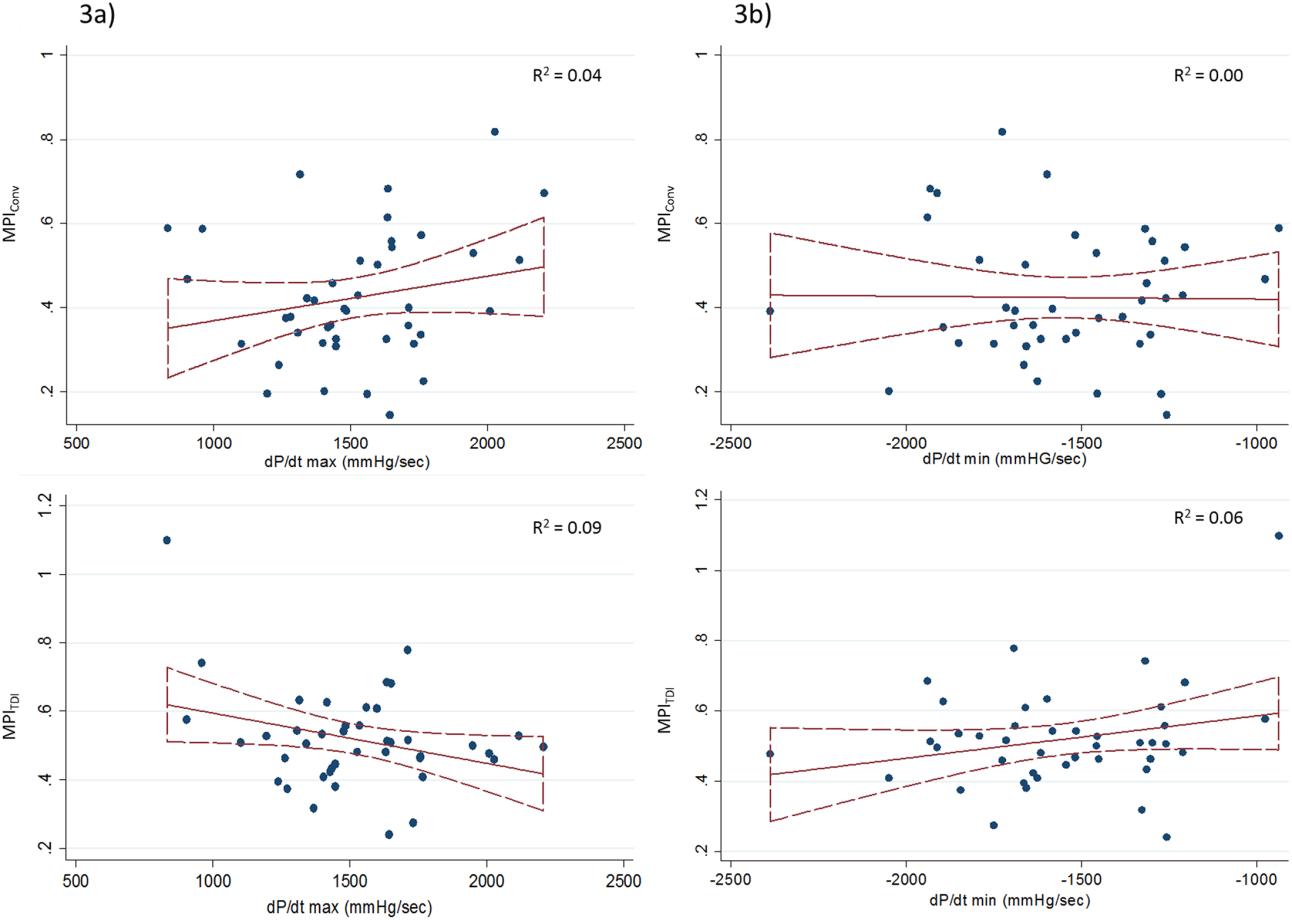
The scatter plots for MPI_TDI_ and MPI_Conv_ according to the values of dP/dt max and dP/dt min, respectively. Depicting the scatter plots and the expected values for MPI_TDI_ and MPI_Conv_ according to the values of dP/dt max (Fig 3a) and dP/dt min (Fig 3b), respectively. Dotted curves indicate 95% confidence intervals. dP/dt max = the rate of LV pressure rise in early systole; dP/dt min = the rate of LV pressure decline in early diastole; MPI_TDI_ = Myocardial Performance Index by TDI M-mode; MPI_Conv_ = Myocardial Performance Index by the conventional method.

The clinical characteristics of the validation cohort undergoing left heart catheterization are displayed in Table 3. There was no association between MPI_Conv_ and dP/dt max (p = 0.18; Table 4 and Fig 3a). The MPI_TDI_ decreased significantly with increasing values of dP/dt max (p = 0.047; Table 4 and Fig 3a), reflecting improved values of the combined cardiac index with improved cardiac contractility. In comparison, there was no association between dP/dt max and GLS (p = 0.20) or LVEF (p = 0.11). The only systolic echocardiographic parameter which displayed improved systolic function with better LV contractility determined by higher dP/dt max was GL Strainrate s (GL Strainrate s: β = -0.021 (-0.039 to -0.003), R^2^ = 0.12, p = 0.025, per 100 mmHg/sec increase).

**Table 3:** Population characteristics in the validation cohort undergoing left heart catheterization (group B, n = 44).

	Validation cohort (n = 44)
Age (years)	64 ± 12
Female gender	12 (29%)
BMI (kg/m^2^)	26.8 ± 4.2
Heart Rate (beats per minute)	68 ± 13
Coronary angiography	
0—vessel disease	14 (33%)
1—vessel disease	12 (29%)
2—vessel disease	6 (14%)
3—vessel disease	10 (24%)
LVEF (%)	53.4 ± 8.4
LAVI (mL/m^2^)	22.4 ± 9.6
E/A ratio	1.0 ± 0.3
DT (ms)	230 ± 50
E/e’	8.8 (7.3–12.0)
LVMI (g/m^2^)	87.3 ± 20.7
GLS (%)	-16.3 ± 3.5
GL SR s (sec^-1^)	-0.8 ± 0.2
GL SR e (sec^-1^)	0.9 ± 0.3
MPI_TDI_	0.54 ± 0.17
MPI_Conv_	0.44 ± 0.17

BMI = Body Mass Index; LVMI = Left Ventricular Mass Index; LVEF = Left Ventricular Ejection Fraction; E = peak transmitral early diastolic inflow velocity; A = peak transmitral late diastolic inflow velocity; DT = deceleration time of early diastolic inflow; e’ = average peak early diastolic longitudinal mitral annular velocity determined by pulsed-wave tissue Doppler imaging from the septal and lateral walls; GLS = global longitudinal strain; GL SR s = global longitudinal systolic strain rate; GL SR e = global longitudinal early diastolic strain rate; MPI_TDI_ = Myocardial Performance Index by TDI M-mode; MPI_Conv_ = Myocardial Performance Index by the conventional method.

**Table 4:** The association between MPI_TDI_ and MPI_Conv_, and invasive and echocardiographic measures of systolic and diastolic obtained from the validation cohort (group B, n = 44).

	MPI_TDI_		MPI_Conv_	
	Beta (95% CI)	*p-value*	Beta (95% CI)	*p-value*
**Invasive measures of systolic and diastolic function:**				
dP/dt max, per 100 mmHg/sec increase	-0.015 (-0.029 to -0.000)	*0*.*047*	0.011 (-0.005 to 0.0263)	*0*.*18*
dP/dt min, per 100 mmHg/sec decrease	0.012 (-0.003 to 0.027)	*0*.*12*	-0.001 (-0.017 to 0.016)	*0*.*94*
tau, per 1 sec increase	-0.013 (-0.073 to 0.046)	*0*.*65*	0.004 (-0.052 to 0.060)	*0*.*89*
**Conventional and novel echocardiographic measures of systolic and diastolic function:**				
LVEF, per 1% increase	-0.743 (-1.338 to -0.148)	*0*.*016*	-0.613 (-1.220 to -0.007)	*0*.*048*
e’, per 1 cm/sec decrease	0.035 (0.018 to 0.053)	*<0*.*001*	0.021 (0.001 to 0.040)	*0*.*026*
E/e’, per 1 increase	0.003 (-0.007 to 0.013)	*0*.*56*	0.002 (-0.008 to 0.011)	*0*.*74*
GLS (%), per 1% decrease	0.021 (0.010 to 0.033)	*0*.*001*	0.009 (-0.004 to 0.022)	*0*.*16*
GL SR s, per 1 sec^-1^ decrease	0.525 (0.306 to 0.745)	*<0*.*001*	0.240 (-0.002 to 0.482)	*0*.*051*
GL SR e, per 1 sec^-1^ increase	-0.481 (-0.618 to -0.343)	*<0*.*001*	-0.361 (-0.511 to -0.210)	*<0*.*001*

dP/dt max = the rate of LV pressure rise in early systole; dP/dt min = the rate of LV pressure decline in early diastole; LVEF = Left Ventricular Ejection Fraction; E = peak transmitral early diastolic inflow velocity; e’ = average peak early diastolic longitudinal mitral annular velocity determined by pulsed-wave tissue Doppler imaging from the septal and lateral walls; GLS = global longitudinal strain; GL SR s = global longitudinal systolic strain rate; GL SR e = global longitudinal early diastolic strain rate; MPI_TDI_ = Myocardial Performance Index by TDI M-mode; MPI_Conv_ = Myocardial Performance Index by the conventional method.

Neither MPI_Conv_ nor MPI_TDI_ were significantly associated with dP/dt min (Table 4 and Fig 3b). With regards to MPI_TDI_ we did, however, find a trend where MPI_TDI_ seemed to increase with increasing values of dP/dt min (lower numerical values)(Table 4 and Fig 3a), reflecting a worsening diastolic function (Table 4 and Fig 3b). In contrast, for MPI_Conv_, if a trend was present, it seemed reversed, as MPI_Conv_ increased with decreasing values of dP/dt min (higher numerical values), reflecting improved diastolic function (Table 4 and Fig 3b). In comparison, neither e’, GL Strainrate e, nor E/e’ were significantly associated with changes in dP/dt min (e’: β = 0.155 (-0.086 to 0.396), R^2^ = 0.04, p = 0.20, per 100 mmHg/sec decrease; GL Strainrate e: β = 0.005 (-0.021 to 0.031), R^2^ = 0.00, p = 0.71, per 100 mmHg/sec decrease; E/e’: β = -0.006 (-0.532 to 0.520), R^2^ = 0.00, p = 0.98, per 100 mmHg/sec decrease).

Like for the dP/dt min, neither MPI_TDI_ nor MPI_Conv_ were significantly associated with tau (Table 4).

### The association between MPI_TDI_ and MPI_Conv_ and established echocardiographic measures of systolic and diastolic function

MPI_TDI_ increased significantly, displaying worsening myocardial function with worsening systolic function determined by LVEF, GLS or GL Strainrate s (Table 4). In contrast, MPI_Conv_ only increased significantly with worsening LVEF (Table 4).

Both MPI_TDI_ and MPI_Conv_ increased significantly with worsening diastolic function determined by e’ or GL Strainrate e (Table 4). Neither MPI_Conv_ nor MPI_TDI_ were significantly associated with E/e’ (Table 4).

Additionally, MPI_TDI_ was defined as increased when above 0.54 for women and 0.56 for men as obtained from group A (Table 2). Furthermore, in group B an impaired systolic function was defined as an LVEF below 50% and impaired diastolic function as e’ below 9 cm/sec (average obtained from the septal and lateral wall by pulsed-wave TDI) and/or an left atrium volume index of 34 mL/m^2^ or above. Applying the cut-offs obtained from group A on group B, resulted in a sensitivity of 26% and a specificity of 100% for the MPI_TDI_ to identify an impaired systolic and/or diastolic function defined by conventional echocardiography (Table 5).

**Table 5:** Diagnostic accuracy of MPI_TDI_ for diagnosing impaired systolic and/or diastolic function in the validation cohort (group B, n = 44).

Cut-offs	Sensitivity	Specificity	PPV	NPV
MPI_TDI_				
> 0.54 for women & > 0.56 for men	26%	100%	100%	3%

Impaired systolic function was defined as an LVEF below 50% and impaired diastolic function as e’ below 9 cm/sec and/or an left atrium volume index of 34 mL/m^2^ or above. MPI_TDI_ = Myocardial Performance Index by TDI M-mode; PPV = Positive Predictive Value; NPV = Negative Predictive Value.

## Discussion

### Normal values of cardiac time intervals stratified by age and gender

Our study is the first to assess the normal values of cardiac time intervals obtained by TDI M-mode in a sample from the general population without cardiovascular disease and cardiovascular risk factors (Table 2). We found that LV function assessed by IVRT, IVRT/ET, and MPI, all decreased with increasing age in both genders (Table 2 and Fig 2). The decrease in LV cardiac function with increasing age as assessed by IVCT and IVCT/ET was only statistically significant in females and not in males (Table 2 and Fig 2). ET decreased with increasing age in both genders; however, this decline was not statistically significant (Table 2 and Fig 2). Other novel echocardiographic parameters detecting subtle myocardial impairments like two-dimensional strain echocardiography[23] and TDI[24] have previously, and in accordance with our results, been demonstrated to detect miniscule LV dysfunction with increasing age despite the absence of any cardiovascular disease or risk factors.

IVCT, ET, IVRT/ET, and MPI all differed significantly between male and female gender (Table 2 and Fig 2). These gender differences remained statistically significant after multivariable adjustment for all other baseline differences between genders (Table 2). Gender differences exist both in novel echocardiographic parameters like two-dimensional strain echocardiography[23] and TDI[24], but also in the cardiac dimensions obtained by conventional echocardiography[21], and in conventional measures of systolic[25] and diastolic function[21,24]. The discrepancy we found, i.e. that women, in general, display better cardiac function than men, appears to be physiologically plausible and in accordance with previous studies[21,23–25].

### The association between MPI_TDI_ and MPI_Conv_ and invasive and echocardiographic measures of systolic and diastolic function

MPI_TDI_ has previously been demonstrated to have better reproducibility[17,18] and to be influenced by fewer physical parameters compared to MPI_Conv_[18]. Additionally, MPI_TDI_ (but not MPI_Conv_) has been proven to be an independent predictor of mortality in the general population[18]. This study is, however, the first to compare the two methods of obtaining MPI with established invasive and echocardiographic measures of systolic and diastolic performance. Our results demonstrate that MPI_TDI_ (but not MPI_Conv_) is significantly correlated with the invasive measure of contractility and systolic function dP/dt max (Table 4 and Fig 3a). The only other echocardiographic measure which was significantly correlated with dP/dt max was GL SR s, which previously has been demonstrated to be the most accurate marker of myocardial contractile function[26]. MPI_TDI_ may, therefore, be an accurate marker of myocardial contractile function as well. In contrast to our results, Tei and colleagues previously demonstrated good correlation between MPI_Conv_ and dP/dt max in a smaller study of patients with IHD[27]. However, in accordance with our results, several studies performed during the last decade which compare MPI obtained by the conventional method as described by Tei and colleagues[10,11] and by TDI, have demonstrated MPI obtained by TDI to be the superior method[17,18,28,29], both when comparing reproducibility[17,18] and their diagnostic and prognostic utilities[18,28,29].

Neither MPI_Conv_ nor MPI_TDI_ were significantly associated with dP/dt min in our study. In comparison, none of the diastolic echocardiographic measures (e’, GL Strainrate e, nor E/e’) were significantly associated with changes in dP/dt min. The lack in correlation between our invasive measure of diastolic function (dP/dt min) and the echocardiographic measures of diastolic function may, therefore, display an inherent limitation in the invasive dP/dt min parameter than in the echocardiographic measures investigated in this study.

Furthermore, MPI_TDI_ increased significantly with worsening systolic function determined by all the echocardiographic measures of systolic function (LVEF, GLS or GL SR s), whereas MPI_Conv_ only increased significantly with worsening LVEF (Table 4). However, both MPI_TDI_ and MPI_Conv_ increased with worsening diastolic function determined by the measures of diastolic function (e’ or GL SR e) (Table 4). Neither MPI_TDI_ nor MPI_Conv_ correlated significantly with E/e’ (Table 4). This may be due to the fact that E/e’ is an echocardiographic measure which correlates well with the LV end diastolic pressure (LVEDP), but is not a sensitive measure of an ailing diastolic function when LVEDP is normal. Likewise, the current guidelines recommend the evaluation of the value e’ (not E/e’) as the first step in the assessment of grading diastolic dysfunction[30]. Additionally, we have previously demonstrated MPI_TDI_ to be independently associated with the conventional echocardiographic measures of systolic and diastolic function (LVEF and e')[7]. In addition, in the present study we demonstrate that MPI_TDI_, but not MPI_Conv_, displays an ailing systolic and diastolic function, as determined by the comparison with 2D-speckle tracking echocardiographic measures (GLS, GL SR s and GL SR e). Our and previous results illustrate that an ailing systolic or diastolic performance can be detected by an increasing value of MPI when assessed by TDI. We found when the MPI_TDI_ was increased in our validation population (group B) above the reference limit obtained from the healthy general population (group A), the probability of identifying an impaired echocardiographic examination, defined by either an impaired systolic and/or diastolic function, was 100%, illustrated by a positive predictive value of 100% (Table 5). Additionally, no one with a normal echocardiography in our validation population had an MPI _TDI_ above our defined reference value illustrated by specificity of 100% (Table 5). Furthermore, MPI_TDI_ seem superior to MPI_Conv_ in the detection of an ailing systolic or diastolic performance regardless of evaluation with conventional echocardiographic, novel echocardiographic or invasive measures.

### Limitations

The inhabitants of Denmark and the study population contributing to the normal values are primarily Caucasian, which limits the generalizability of our findings to other general populations with another race composition.

Our invasive validation population was small, comprised only 44 individuals, which limits our study. Nevertheless, this was a larger study group than can be found in previous studies which evaluate the association of cardiac time intervals with invasive measures of systolic and diastolic function[27,31].

The echocardiograms in group A and B were obtained and analyzed with almost a decade apart why the methods of assessing systolic and diastolic function differ between the two cohorts. These differences, together with the fact that group B consists of patients suspected of ischemic heart disease who underwent coronary angiography and group A consist of healthy participants from the general population, limits the comparability between the two groups. However, the aim of the present study was not to compare the two groups but rather to define normal values of cardiac time intervals obtained by TDI M-mode from the general population without known cardiovascular disease and to evaluate the association of MPI_TDI_ and MPI_conv_ with established invasive measures of systolic and diastolic function.

## Conclusion

The normal values of cardiac time intervals deteriorated with increasing age and differed between genders, displaying that woman, in general, exhibit better cardiac function. MPI_TDI_ (but not MPI_Conv_) is significantly associated with most invasive and established echocardiographic measures of systolic and diastolic function.
